# Diversity begets diversity: A global perspective on gender equality in scientific society leadership

**DOI:** 10.1371/journal.pone.0197280

**Published:** 2018-05-30

**Authors:** Dominique A. Potvin, Emily Burdfield-Steel, Jacqueline M. Potvin, Stephen M. Heap

**Affiliations:** 1 School of Science and Engineering, University of the Sunshine Coast, Hervey Bay, Queensland, Australia; 2 Centre of Excellence in Biological Interactions, Department of Biological and Environmental Science, University of Jyväskylä, Jyväskylä, Finland; 3 Department of Women's Studies and Feminist Research, The University of Western Ontario, London, Ontario, Canada; Indiana University Bloomington, UNITED STATES

## Abstract

Research shows that gender inequality is still a major issue in academic science, yet academic societies may serve as underappreciated and effective avenues for promoting female leadership. That is, society membership is often self-selective, and board positions are elected (with a high turnover compared to institutions)—these characteristics, among others, may thus create an environment conducive to gender equality. We therefore investigate this potential using an information-theoretic approach to quantify gender equality (male:female ratios) in zoology society boards around the world. We compare alternative models to analyze how society characteristics might predict or correlate with the proportion of female leaders, and find that a cultural model, including society age, size of board and whether or not a society had an outward commitment or statement of equality, was the most informative predictor for the gender ratio of society boards and leadership positions. This model was more informative than alternatives that considered, for instance, geographic location, discipline of study or taxonomic focus. While women were more highly represented in society leadership than in institutional academic leadership, this representation was still far short of equal (~30%): we thus also provide a checklist and recommendations for societies to contribute to global gender equality in science.

## Introduction

Gender equity in academic science is yet to be achieved in most institutions and societies worldwide. Although it is clear that the lack of gender diversity and the bias against women that exists in science is detrimental to productivity, innovation and job satisfaction [1, 2], attempts to remedy these issues have met resistance. That is, while there is a trend for nations and institutions to close the wage gap, update hiring practices and implement support systems, the reality is that women are still highly underrepresented in many scientific fields [3, 4].

Despite trends for increasing female enrolment in post-graduate science [5], there is still a significant gender disparity at higher levels of academia (a phenomenon known as hierarchical segregation or “the leaky pipeline”). The reasons for this disparity are the subject of increasing research in multiple disciplines, yet these studies have tended to neglect the part of scientific societies. Often considered as a merely tangential part of academic life, scientific societies play a large role in supporting students and researchers through the provision of grants, conferences, and journal publications. They also often help to unite geographically distant researchers within a field, provide mentors or role models for early career academics, and have the potential to close gaps between career stages by bringing people together with a common scientific goal. Possibly most importantly, societies provide opportunities for networking, both formally and informally. This is particularly valuable for women academics, since it has been shown that women can often feel excluded or isolated from traditional academic networks [6, 7] while also needing to demonstrate higher involvement in such networks in order to achieve promotions [8]. Thus, the scientific society remains one facet of academic life that is not only under studied, but may also have the capacity to support women in their careers, as well as promote necessary systemic and intrinsic changes throughout science.

So, what then is the role of the scientific society in helping rectify gender inequity? Unlike academic institutions, modern scientific societies do not implement a hiring process through a committee. While a few societies require an internal reference or nomination for new members, joining is more often a process of self-selection (one simply chooses to become a member by enrolling and possibly paying a membership fee). Similarly, leadership positions in these societies are often self-nominated and/or elected by the rest of the society membership—including a high number of student members and early career researchers—thus making the process of "climbing the ladder" in a society very different than in an academic institution. Furthermore, the turnover of leadership positions is often relatively high: unlike academic institutions, there is usually no tenure of a position such as society president or treasurer. These factors may mean that the demographic make-up of society leadership is visibly different than that of academic institutions. In turn, these processes of election, self-nomination and frequent turnover may also promote higher levels of diversity in society leadership, and may thus help change the culture of science.

We might expect (or hope), then, that processes of self-selection, nomination and electoral promotion might be more conducive to promoting diversity of all kinds than decisions made by committee or institutional assessment, yet we do not know whether this is the case. Of course, it may be that societies, due to their informal nature and lack of accountability to external parties or institutional policies, might demonstrate high levels of gender inequality in leadership, excluding women from one more aspect of academic life [9]. Our study, therefore, aims to quantify gender equality (defined as proportionally equal representation of men and women) on the councils and boards of academic scientific societies—specifically in zoological sciences. We do this by examining the representation of gender on the governing boards or councils of a number of zoological societies, and also consider the gender of those in primary leadership positions (president, vice president, secretary and treasurer). We are using gender representation in leadership boards as a measurement for a few reasons. Firstly, it is a simple and quantitative measurement that can lend itself to statistical analysis. Second, it is still likely an indication of broader gender equality within an organization: previous studies have found that organizations with female leaders tend to be more equitable overall in terms of gendered attitudes and treatment [10, 11]. Finally, revealing organizational structures or variables that may play a role in limiting women’s leadership may also indicate where the barriers to female opportunity lie overall in these organizations.

We further aimed to identify the characteristics of societies that might be correlated with gender equality, as being able to distinguish specific societal traits that are associated with gender equality might help us make targeted recommendations for promoting women in scientific leadership. The first of these variables was an obvious, outward commitment to equality through affirmative action, constitutional wording, or any societal roles dedicated to gender equality. We expect that such commitments will have a positive effect on promoting gender diversity. We also considered the geographical scope of the society, as biases in the perception of whether science is gendered tend to be predicted by national culture [12, 13]. Thus, we might predict that societies with a more international reach may be more likely to demonstrate gender equality in leadership, and that gender representation varies with the geographic region of a society. We also take into account the age of a society, a factor that has been found to contribute towards gendered biases in academic institutions (with older institutions generally demonstrating more bias than younger institutions) through institutional culture [14–16]. Finally, we investigate whether gender equality differs based on the scope of the society’s interests, either being discipline based (e.g. ecology, evolution) or taxon-based (e.g. ornithology, herpetology). Taxon-based societies are frequently born out of groups of field naturalists—a culture that flourished during an era when gender discrimination was commonplace; whether this culture and subconscious bias remains can therefore be evaluated.

## Methods

### Data collection

We concentrated our study on scientific, academic professional societies: we did not consider corporations, private organizations, lobbying or political groups, or project-based organizations focused primarily on fundraising rather than science (e.g. trusts). The society had to be open (membership was not selective or hiring-based, i.e. "research centres"). We limited our data collection to societies with a focus on living animals, in order to keep the study within our own field of expertise. This included societies centered around either taxanomic groups limited to the level of Class (not below, e.g. mammal societies, ornithological societies but not raptor societies), and disciplines (e.g. animal behaviour societies, ecological societies—or other societies with zoologists as members). We did not, however, consider associations based solely on animal keeping or husbandry—as mentioned above, the society must have a scientific research focus. In order to obtain data, and keeping these guidelines in mind, we completed two major Google searches. The first was for taxon-based societies, which we looked for using the following search terms: "society" OR "association" OR "union" AND "bird" OR "ornithol*" OR "herpetol*" OR "reptile" OR "amphibian" OR "entomol*" OR "fish" OR "icthyol*" OR "insects" OR "invertebrates" OR "marine" OR "mammal" OR "mammol*" OR "zoology". For discipline-based societies, we used the following search terms: "society" OR "association" OR "union" AND "conservation" OR "ecology" OR "evolution" OR "ethology" OR "behaviour" OR "cognition" OR "genetics" OR "biology" OR "physiology" OR "morphology" OR "naturalist" NO "human" NO "plant".

In order to make sure data was reliable, we used only societies that had a current website (as of April-May 2016), either in English or easily translatable through online translation platforms. We then collected the following information from each society: the number of members that made up the main executive board or committee, the number of women on those committees, and the gender of each of the people in the following positions: President (or equivalent, e.g. Director), Vice-President, Secretary and Treasurer. We also collected data regarding the society's founding year, the country in which the society is based, the main focus of the society and its geographic scope. All data collected can be found in S1 Table in the Supplementary materials. We also then completed a thorough search throughout the website to find any evidence of a statement, committee or other form of affirmative action program that implies that the society is dedicated to increasing diversity or improving gender equality. Typically, these statements could be found in the society's written or published constitution or bylaws, or was presented clearly on the website. Once all pages and documents from the website were searched, if no statement or committee was found, the society was considered not to have such a statement. While we realize this may not in fact be true, a statement is not particularly useful if it is hidden or can only be seen by certain members of a society. We thus termed this variable "visible statement or action" and categorized it as present or absent.

We note that our determination of gender was non-intrusive, and was therefore based on photographs, names or, in the best-case scenarios, descriptions. In cases where names were difficult to determine, a search for the researcher based on manuscripts they had written in the field allowed for more information to be gathered for a decision on gender to be made through personal or institutional websites. The gender being reported is that of the performative gender of the person rather than the biological sex of the person. This also meant that those identifying as gender non-binary could only be included if this identification was specifically stated, however we did not come across this scenario in the course of our research.

We want to state that while we are very interested in the ideas of intersectionality and other forms of diversity in academia, we chose to investigate gender only, due to the availability of data, the amount of previous research on the topic, and our own areas of expertise. Similar studies of societies may therefore be completed on other aspects of diversity including race, age, ethnicity, sexuality, religion and income level. We would encourage societies that might be concerned about their lack of diversity and representation, or that would like to improve these aspects to do health checks—as described below—of their membership and leadership with the goal of implementing measures such as affirmative action statements and programs to improve the quality of their science and their global impact by increasing their diversity and representation of all scientists.

### Statistics

We investigated a) the proportional representation of women on a society board (FemProp), b) the presence of a woman in the position of society president or equivalent (FemExec), and c) the number of women in leadership positions (president, vice-president, secretary, treasurer; FemLead) using an Information-Theoretic approach [17–20]. Drawing from the literature [12–16], we established five candidate models that may explain variation in these variables (Tables 1 and 2) and compared their explanatory potential using their corrected Akaike Information Criterion (AICc). The first of these models attempted to reflect broad aspects of each society board’s social network, in terms of its size and gender representation (Network). A second model aims to capture the organizational or workplace culture of the society according to the social network of their boards (as above), the society age and whether there are any commitments to promoting equality (Culture). Third, we considered a model that represents the scope of the society in terms of its disciplinary focus and regional scale (Scope). Fourth, we tested geographic variation by including a model consisting of each society’s region of origin and the scale of its reach (Geography). Finally, we included a historical model that included only each society’s age (History).

**Table 1:** List of predictor and response variables used in the models.

**Predictor Variable**	**Description**
Board Size	Number of people on the society's academic board;
	square-root transformed for normality
Female Board Representation	Number of females on the board;
(not incl. in FemProp)	square-root transformed for normality
Female Leadership Count	Count of females in the roles of executive, vice-executive, secretary and treasurer; executive not counted in FemExec model
(not incl. in FemLead)	
Society Age	Year of society founding
Statement of Equality	Binary for the presence of a visible statement of equality online
Discipline	Whether society has a disciplinary (e.g. conservation, evolution, ecology) or taxonomic (e.g. herpetology, ornithology, mammalian) focus
Scale	Whether society operates on a national, continental or international scale
Region	Continent in which the society was founded
**Response Variable**	**Description**
Proportional Female Board Representation (FemProp)	x out of every n board members are female (i.e. female board member = success, male member = failure)
Female Executive (FemExec)	Binary for the presence of a female in the executive leadership position (e.g. president, chairperson)
Female Leadership Count (FemLead)	Count of females in the roles of executive, vice-executive, secretary, treasurer

**Table 2:** The different explanatory models being compared. Dark grey indicates the presence of a predictor variable in the model. Light grey indicates variables that are included in some tests, but not others (i.e. when they are the response variable).

	Models						
Predictor Variable	Society Culture	Board Network	Society Scope	Geography	History	Full	Null
Board Size							
Female Board Representation (not incl. FemProp)							
Female Leadership Count (not incl. FemLead)							
Society Age							
Statement of Equality							
Discipline							
Scale							
Region							

We tested the fit and suitability of a global model that included all predictor variables, after removing societies that had missing data. We used GLM’s with a binomial distribution and logit link for both FemProp and FemExec models, whilst a GLM with poisson distribution and log-link was used for the FemLead model. We used pseudo-r^2^ (r^2^_p_) as a measure of the proportion of variance a model could explain [1 –(model deviance/null model deviance)]. The AICcmodavg package [21], running on R 3.3.1 calculated the QAICc (due to overdispersion) for FemProp models, and the AICc for FemExec and FemLead models. Furthermore, we calculated the natural model average estimates for each predictor.

## Results

**Fig 1:**
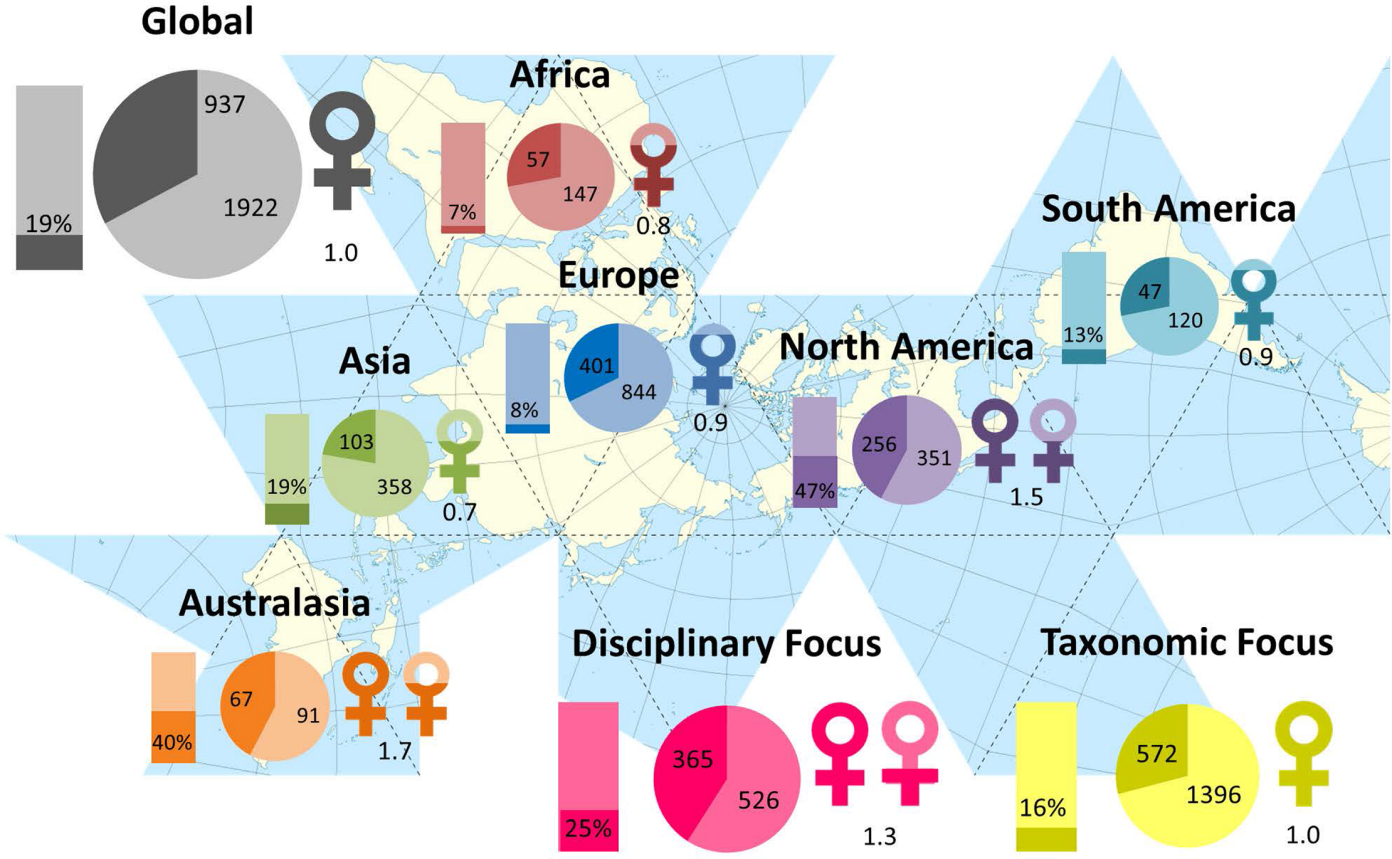
Raw numbers of women on zoological society boards worldwide. Pie charts show the number of men and women on the boards of scientific committees across different geographic regions, and society focus (i.e. disciplinary or taxanomic). Darker colours denote women, lighter colours men. Bar graphs show the proportion of societies that have diversity statements (to the nearest percentage). Female symbols represent the average count for the number of women fulfilling leadership roles within a society (president, vice-president, secretary, treasurer) to the nearest decimal place. For example, in Asia: 19% of societies have diversity statements, 358 board positions are taken up by men and 103 by women, and the average number of women in senior board positions is 0.7 (out of four possible positions).

We obtained the minimum required data from 202 separate scientific societies. Raw data collected from each society (as of April-May 2016) can be found in S1 Table. In total, 68 societies were discipline based and 134 were taxon-focused; 142 were national societies, 27 were continental and 33 were international (multiple continents). In total, only 39 societies (19.3%) had a visible statement or commitment to diversity and/or gender equality. The mean age of societies was 58 years, with the oldest society (Société Entemologique de France) being founded in 1832 (184 years old), and the youngest societies (multiple) founded in 2012 (4 years old). Fig 1 demonstrates the percentages of women and men on society boards by geographic area.

### Female board representation

**Fig 2:**
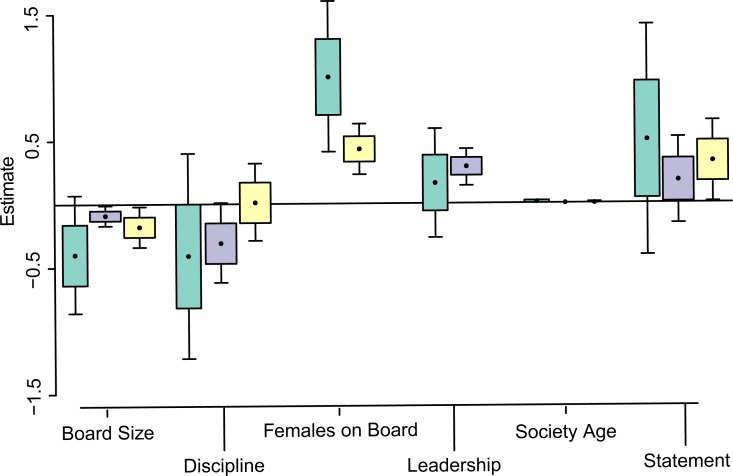
Natural model averages for the predictors of the society culture model, including also the disciplinary focus factor, for FemExec (woman in a leadership position: Green), FemProp (proportional female board representation: Purple) and FemLead (female leadership count: Yellow) tests. Estimates are given by the points, boxes indicate standard errors and whiskers indicate 95% confidence intervals.

Factors of society culture explained more variation in female board representation than factors based on history, geographical scale or disciplinary scope (Table 3). Specifically, female board representation tends to be higher in societies with smaller board sizes and with more women in leadership positions (Fig 2; S2 Table). Furthermore, we have some confidence that taxonomic societies have less female representation than disciplinary societies, yet models that include discipline (Full, Scope) are substantially less informative than the Cultural model and the 95% confidence interval for the predictor estimate crosses zero (Fig 1).

**Table 3:** The set of models used to explain variation in the proportion of females on the boards of biological societies (N = 188).

Model	K	QAICc	Δ QAICc	QAICcWt	∑ Wt	QLL	r^2^_p_
Culture	6	364.10	0.00	0.97	0.97	-175.82	0.16
Full	14	371.37	7.27	0.03	1.00	-170.47	0.21
Network	4	383.46	19.36	0.00	1.00	-187.63	0.15
History	3	388.44	24.34	0.00	1.00	-191.15	0.22
Scope	5	404.92	40.82	0.00	1.00	-197.30	0.07
Geography	9	405.30	41.20	0.00	1.00	-193.18	0.10
Null	2	414.67	50.57	0.00	1.00	-205.30	< 0.01

Full Model Residual Deviance = 508.52 on 175 df

Culture Model Residual Deviance = 538.98 on 183 df

### Female executive representation

Society culture provided the most informative model for explaining the presence of females in the role of president (Table 4). Of the cultural factors considered, we can be confident that female presidents are far more likely to be found in societies with greater female board representation. There is also a very small tendency for older societies to not have female presidents. Other cultural factors do not appear to have any consistent effect on predicting female executives (Fig 2; S3 Table).

**Table 4:** The set of models used to explain variation in the presence of females as chief executive of biological societies (N = 186).

Model	K	AICc	Δ AICc	AICcWt	∑ Wt	LL	r^2^_p_
Culture	6	191.55	0.00	0.94	0.94	-89.54	0.11
Full	14	197.37	5.81	0.05	1.00	-83.45	0.17
History	2	202.56	11.00	0.00	1.00	-99.25	0.02
Network	4	205.66	14.11	0.00	1.00	-98.72	0.09
Scope	4	212.66	21.11	0.00	1.00	-102.23	0.06
Geography	8	218.05	26.49	0.00	1.00	-100.64	0.07
Null	1	219.04	27.49	0.00	1.00	-108.51	< 0.01

Full Model Residual Deviance = 166.91 on 172 df

Culture Model Residual Deviance = 179.08 on 180 df

### Female leadership representation

The model for society culture was clearly the most informative in explaining the number of female leaders in zoological societies (Table 5). We can be confident that the number of women in leadership positions increases with the number of women on the board and with the presence of a statement of equality (Fig 2; S4 Table). Additionally, we have some confidence that the number of women in leadership positions decreases with the total size of the board, although the confidence interval for this factor slightly crosses zero (Fig 2).

**Table 5:** The set of models used to explain variation in the number of females in leadership positions of zoological societies (N = 188).

Model	K	AICc	Δ AICc	AICcWt	∑ Wt	LL	r^2^_p_
Culture	5	483.65	0.00	0.99	0.99	-236.66	0.13
Full	13	492.21	8.56	0.01	1.00	-232.06	0.18
History	2	507.46	23.81	0.00	1.00	-251.70	0.01
Network	3	512.52	28.87	0.00	1.00	-253.20	0.10
Geography	8	529.74	46.09	0.00	1.00	-256.49	0.09
Scope	4	535.00	51.35	0.00	1.00	-263.40	0.03
Null	1	535.03	51.38	0.00	1.00	-266.50	< 0.01

Full Model Residual Deviance = 169.01 on 175 df

Culture Model Residual Deviance = 178.21 on 183 df

## Discussion

Of all our predictive models, we identified that of 'society culture' as clearly the most informative for explaining female representation on the boards of zoological societies. Specifically, societies with smaller boards have greater female representation on the board as a whole, and such societies are more likely to elect female leaders. Furthermore, the likelihood for women to occupy leadership positions is improved by a visible statement of equality. Whilst there is a very small tendency for taxonomic and older societies to have fewer women on their boards, we have far less confidence in how meaningful these trends are: although we note that founding year is also a predictive factor for gender equality in leadership at institutions [22] Thus, societies with a high proportion of female board members, societies with women in leadership roles and societies with a statement of gender equality all tend to be the same societies: these variables are good indicators of each other.

Our results suggest that the internal culture (as defined or represented by the quantitative variables of broader board gender representation, society age along with any diversity, equality or anti-discrimination commitments) and structure of a society is more influential in predicting female representation than whatever biases are inherited from geographic region, taxonomic focus or historical precedent. Thus, attention given directly to aspects of the society board may provide effective means for overcoming obstacles that stem from broader cultural aspects. Prior research has shown that exposure to women in leadership positions can reduce the negative stereotypes surrounding women in positions of authority [23]. So the presence of at least one woman in a prominent position within the society could be promoting the election of other women. Interestingly, this pattern has not been reflected in previous research examining academic workplaces: the number of women leaders has only recently been found to influence patterns of gender equity for institutional leadership [24–26]. It is possible this discrepancy comes from the differing patterns of selection between academic societies and other types of institution. However, since our data shows only existing patterns we can merely speculate as to the underlying causation.

In addition, our finding that smaller boards tend to be more gender-equal is interesting, and may demonstrate that smaller boards may be less intimidating for senior female members–however this is speculation. While there is plentiful qualitative evidence to suggest that the presence of more senior women can make organizations more welcoming and accessible to female researchers and leaders [10], and that mentoring can significantly increase the retention and career progression of female junior researchers [27], quantative data on the effects of female leaders in academia on diversity remain scarce. Our study provides quantitative data to echo previous qualitative research showing that female leadership and the proportion of women in an organization are positively linked [11], although of course we can only speculate as to the causation.

Although female leadership in zoological societies is still limited (e.g. only 30% of societies sampled had at least 50% of leadership positions filled by women; Fig 1), it does supercede that of female scientific leaders (deans and presidents) in academic institutions [5–25%; 28]. The reasons for this limited representation are far beyond the scope of this paper to discuss fully, as the models we considered are so broad as to neglect much of the finer scale social and organizational patterns that are likely at work. Furthermore, our models were not able to fully explain the variability in gender representation in societies, and thus further research into more detailed aspects of culture or bias is warranted. For instance, research to date would suggest that female leadership can be affected by intrinsic biases that unconsciously affect the way we view and interact with certain groups [29], and which often work against women seeking positions of authority. Research has also shown that leadership traits are more readily associated with men than women [30, 31], and this, along with other negative stereotypes associated with women, could hinder their ability to be elected into leadership roles. Paradoxically, female and minority leadership is often effective in contradicting such associations and breaking negative stereotypes, changing attitudes of employees and members, thus leading to broader acceptance of women leaders in general—but only if they are able to attain top positions in the first place [32, 33].

With regards to equality statements and female representation, it is impossible to know in which direction causality lies. That is, it is unclear whether such statements increase the number of women in society leadership positions, whether women leaders are influential in producing such documents, or whether some other factor is involved. At institutions, it has been found that government or national gender equity or anti-discrimination policies have little or no bearing on the number of female academics in leadership positions [34], suggesting that statements or policies alone are inadequate for implementing real change, and it may be that the visibility or accountability of such statements are just as important as the statements themselves. However, it does not mean that they do not form an important part of the culture of a body that is committed to equality. In any case, increasing female leadership and including a constitutional commitment to equality (such as through a statement of support or objective quotas) are both actions that can be directly implemented by scientific societies. They can thus be part of a larger 'health check' that can be performed by societies to assess their current state of affairs in terms of gender equality. Gender-equality 'health checks' can be useful not only for understanding the current situation of a society, but also provide targets and pathways to increasing female representation and female involvement in science. Toolkits and health checklists for gender equality can be found in many fields, and are based on evidence–both quantitative and qualitative–in previous literature as to effective ways for promoting and maintaining gender equality [35]. Here, we have modeled such a health check list on one found in Ferber (35). We have chosen a few of these rules that apply to scientific societies to provide an overview. Our results show that the culture of a society is important to promoting gender equality, and although we are unable to show the influence of the specific actions included below, this checklist includes commonly researched actions that have been found to contribute to a more gender-balanced culture [34]. Academic and scientific societies can check their "gender equity health" easily via the checklist outlined in Table 6.

**Table 6:** Health checklist for scientific societies aiming for gender equality.

Gender Equality checklist
Mission statement, vision statement or constitutional statement about inclusion and diversity
Administrative support in the form of a committee or member dedicated to diversity and equality
Written expectations for appropriate behaviour at meetings and conferences
Commitment to keeping demographic data
Commitment to keeping data on transgressions
Support systems for women in the form of formal or informal mentorship or references
Equitable distribution of resources
Gender neutral restrooms at conferences and meetings
Performance or other reviews that value inclusivity
Grievance policies and procedures
Response systems and processes for harassment or discrimination
Objective criteria and/or blind reviewing for conference papers and awards
Family-friendly policies during conferences and meetings
Commitment to identifying and rectifying societal-based intrinsic biases
Communication about inclusion, diversity and equity to wider membership and during recruitment
A knowledge base of feminist/social justice issues in the membership or through societal resources
Support for professional development or training in diversity
Safety considerations for online and conference interactions
Valuing scholarship on diversity issues within the society
Inclusivity as a step in decision-making processes by board members
Commitment to keeping a history of efforts for inclusivity and diversity by the society

This health checklist can be used as a guide, or may even help to inform Key Performance Indicators for societies and groups, and is meant to be a malleable resource that can be tailored by societies according to their particular strategies for assessing their performance or maintaining accountability. Including a health checklist for gender equality in this manuscript is not intended to shame societies that appear to still be heavily male-biased: as mentioned, there are many, varied reasons for inequality. For example, because society leadership is often made up of more experienced academics, societal committees can (like academic departments) be demonstrative of the 'leaky pipeline' problem whereby women are being lost at a high rate during the early phase of the academic career [36]. This may be compounded by the reported higher burden on existing female faculty to serve on boards and committees in order to meet diversity requirements [37], leaving senior female scientists with less time to serve in societies compared to their male colleagues. Furthermore, societies that appear to have higher numbers of women leaders may in fact still harbor intrinsic biases, resulting in women leaders experiencing other forms of sexism [38–40]. Finally, societies with large student involvement may be under the impression that the society as a whole is very gender equal: in the biological sciences, women make up almost 50% of PhD graduates globally (National Science Foundation, 2014). This apparent equality can present a type of façade and result in apathy or non-action in terms of pushing for diversity and gender equity in the society as a whole, particularly concerning leadership [41, 42]. It is not our intention to go into the details and nuances of these particular issues (indeed, the underlying mechanisms for these effects vary greatly according to geographic area, discipline, politics and economy), but to simply highlight them as possible causes for the current state of gender equity across scientific societies.

Rather, we hope to present this data as a way to inspire action by zoological (and other) societies around the world in order to increase the visibility and influence of female members and scientists. Because societies do not generally select leaders "behind closed doors", nor by a powerful few (board members are nominated and elected by all society members) societies allow much more freedom than university departments in how they help women become leaders in their field (for better or worse). The health checklist we have provided may help societies identify themselves as being one of the following: 1) Exclusionary, whereby societies are discriminatory and restrictive; 2) Club-like, preserving a masculine culture while admitting "token" female leaders (that is, women paraded purely as symbols of diversity, often without full inclusion into leadership processes or decisions) without full commitments to equality and diversity; 3) Complying, whereby the society overtly removes discrimination but does not change their mission, structure or culture; or 4) Affirming, whereby the commitment to eliminating discrimination is coupled with changes in the culture that provide support for minority groups and the implementation of policies ensuring equity and diversity [35].

Of course, it is our hope that equity can be achieved throughout the sciences via a number of means, and we encourage societies–including all officeholders and members—to do their part in helping women into leadership positions. To this end, we suggest putting into action some key components of the health checklist:

1. Include a visible (i.e. obvious on the website or in the constitution) mission/vision statement or other commitment to equality and diversity within the organization, which especially includes a non-discrimination clause regarding elected officials and members.
2. Collect data on membership and leadership of the society each time an annual general meeting is held, and present these numbers publicly. This will help societies maintain accountability with members, the academy and the public.
3. Have a specific protocol for the reporting of, and responding to, discrimination and harassment within the society.
4. Be explicit about support for women (and other minority groups). This can occur formally (e.g. providing grants to women, rewording constitutions to be gender-neutral) or informally (e.g. rewarding inclusive behaviour when considering award candidates or nominations, encouraging women at general meetings to take up leadership positions or to speak, and having gender-neutral, non-exclusive or family-friendly social activities).

It is well-documented that organizations with more diverse and equitable representation, especially in leadership, are not only more inclusive and supportive of all members [10, 11] but often more productive and innovative [43, 44]. Furthermore, increased female representation may be beneficial to smaller societies, which may struggle to recruit new members, and fill active board positions by changing what may otherwise be an intimidating environment compared to a visibly inclusive one [3, 45]. By being able to identify particular characteristics that promote (or are associated with) this diversity, we hope that this study gives tools for zoological and other scientific societies to not only keep a finger on the pulse of equality, but also provide them with tools for helping more women feel included in scientific leadership. This step will hopefully help bring science toward achieving equity as a whole, by bringing women and men to the forefront of science as the elected leaders of their fields and thereby influencing academies, institutions and funding bodies.

## Supporting information

S1 Table Database. All Raw Data collected from each society included in the study.
(XLSX)

S2 Table Natural model averages for variables in predicting the proportion of females on society boards. (DOCX)

S3 Table Natural model averages for variables in predicting the presence of a female executive on society boards. (DOCX)

S4 Table Natural model averages for variables in predicting the number of female leaders on society boards. (DOCX)
