# Supplementary material for: Diversity begets diversity: A global perspective on gender equality in scientific society leadership
Source: PLoS One. 2018 May 30;13(5):e0197280. doi: 10.1371/journal.pone.0197280 (PMC5976142; doi:10.1371/journal.pone.0197280)
Supplement: S2 Table — (DOCX) [file pone.0197280.s002.docx]

**Supporting Information**

S2 Table. Natural model averages for variables in predicting the proportion of females on society boards.

| **Factor** | **Estimate** | **SE** | **Lower CI** | **Upper CI** | **w** |
| --- | --- | --- | --- | --- | --- |
| Society Age | 0.00 | 0.00 | 0.00 | 0.00 | 1.00 |
| Leadership | 0.29 | 0.07 | 0.14 | 0.43 | 1.00 |
| Board Size | -0.09 | 0.04 | -0.17 | -0.01 | 1.00 |
| Statement | 0.18 | 0.17 | -0.16 | 0.52 | 1.00 |
| Discipline | -0.31 | 0.16 | -0.62 | 0.01 | 0.03 |
| National vs International Scale | 0.00 | 0.20 | -0.39 | 0.40 | 0.03 |
| National vs Continental Scale | -0.13 | 0.27 | -0.66 | 0.40 | 0.03 |
| Africa vs Europe | 0.17 | 0.32 | -0.46 | -0.80 | 0.03 |
| Africa vs N. America | 0.45 | 0.35 | -0.24 | 1.15 | 0.03 |
| Africa vs Asia | -0.05 | 0.37 | -0.78 | 0.68 | 0.03 |
| Africa vs Australasia | 0.38 | 0.42 | -0.45 | 1.20 | 0.03 |
| Africa vs S. America | -0.09 | 0.44 | -0.94 | 0.77 | 0.03 |
