# Supplementary material for: Diversity begets diversity: A global perspective on gender equality in scientific society leadership
Source: PLoS One. 2018 May 30;13(5):e0197280. doi: 10.1371/journal.pone.0197280 (PMC5976142; doi:10.1371/journal.pone.0197280)
Supplement: S3 Table — (DOCX) [file pone.0197280.s003.docx]

Supporting Information

S3 Table. Natural model averages for variables in predicting the presence of a female executive on society boards.

| **Factor** | **Estimate** | **SE** | **Lower CI** | **Upper CI** | **w** |
| --- | --- | --- | --- | --- | --- |
| Society Age | 0.01 | 0.01 | 0.00 | 0.02 | 1.00 |
| Leadership | 0.16 | 0.22 | -0.27 | 0.59 | 1.00 |
| Females on Board | 1.00 | 0.30 | 0.41 | 1.60 | 1.00 |
| Board Size | -0.40 | 0.24 | -0.86 | 0.07 | 1.00 |
| Statement | 0.50 | 0.46 | -0.41 | 1.41 | 1.00 |
| Discipline | -0.41 | 0.41 | -1.22 | 0.40 | 0.05 |
| National vs International Scale | 0.88 | 0.52 | -0.14 | 1.90 | 0.05 |
| National vs Continental Scale | 0.75 | 0.57 | -0.37 | 1.86 | 0.05 |
| Africa vs Europe | -0.69 | 0.77 | -2.20 | 0.81 | 0.05 |
| Africa vs N. America | -0.66 | 0.87 | -2.37 | 1.06 | 0.05 |
| Africa vs Australasia | -0.64 | 0.99 | -2.57 | 1.29 | 0.05 |
| Africa vs Asia | -1.72 | 1.05 | -3.78 | 0.35 | 0.05 |
| Africa vs S. America | -1.84 | 1.32 | -4.43 | 0.75 | 0.05 |
