# Supplementary material for: Diversity begets diversity: A global perspective on gender equality in scientific society leadership
Source: PLoS One. 2018 May 30;13(5):e0197280. doi: 10.1371/journal.pone.0197280 (PMC5976142; doi:10.1371/journal.pone.0197280)
Supplement: S4 Table — (DOCX) [file pone.0197280.s004.docx]

Supporting Information

S4 Table. Natural model averages for variables in predicting the number of female leaders on society boards.

| **Factor** | **Estimate** | **SE** | **Lower CI** | **Upper CI** | **w** |
| --- | --- | --- | --- | --- | --- |
| Society Age | 0.00 | 0.00 | 0.00 | 0.01 | 1.00 |
| Females on Board | 0.43 | 0.10 | 0.23 | 0.63 | 1.00 |
| Board Size | -0.18 | 0.08 | -0.34 | -0.02 | 1.00 |
| Statement | 0.33 | 0.16 | 0.01 | 0.65 | 1.00 |
| Discipline | 0.01 | 0.16 | -0.29 | 0.32 | 0.01 |
| National vs International Scale | 0.08 | 0.20 | -0.31 | 0.47 | 0.01 |
| National vs Continental Scale | 0.38 | 0.21 | -0.03 | 0.80 | 0.01 |
| Africa vs Europe | 0.08 | 0.35 | -0.61 | 0.77 | 0.01 |
| Africa vs N. America | 0.25 | 0.38 | -0.50 | 0.99 | 0.01 |
| Africa vs Australasia | 0.42 | 0.40 | -0.37 | 1.20 | 0.01 |
| Africa vs Asia | -0.24 | 0.42 | -1.07 | 0.59 | 0.01 |
| Africa vs S. America | 0.08 | 0.44 | -0.77 | 0.94 | 0.01 |
